# Neuropeptide VGF Promotes Maturation of Hippocampal Dendrites That Is Reduced by Single Nucleotide Polymorphisms

**DOI:** 10.3390/ijms18030612

**Published:** 2017-03-11

**Authors:** Joseph Behnke, Aneesha Cheedalla, Vatsal Bhatt, Maysa Bhat, Shavonne Teng, Alicia Palmieri, Charles Christian Windon, Smita Thakker-Varia, Janet Alder

**Affiliations:** Department of Neuroscience and Cell Biology, Rutgers Robert Wood Johnson Medical School, Piscataway, NJ 08854, USA; jabehnke3@gmail.com (J.B.); aneesha.cheedalla@gmail.com (A.C.); bhattvy@rwjms.rutgers.edu (V.B.); maysa93@gmail.com (M.B.); slt119@rwjms.rutgers.edu (S.T.); aliciajpalmieri@gmail.com (A.P.); cwindon82587@gmail.com (C.C.W.); varia@rutgers.edu (S.T.-V.)

**Keywords:** neuropeptide, dendrite, single nucleotide polymorphism, spine, synapse, hippocampus

## Abstract

The neuropeptide VGF (non-acronymic) is induced by brain-derived neurotrophic factor and promotes hippocampal neurogenesis, as well as synaptic activity. However, morphological changes induced by VGF have not been elucidated. Developing hippocampal neurons were exposed to VGF through bath application or virus-mediated expression in vitro. VGF-derived peptide, TLQP-62, enhanced dendritic branching, and outgrowth. Furthermore, VGF increased dendritic spine density and the proportion of immature spines. Spine formation was associated with increased synaptic protein expression and co-localization of pre- and postsynaptic markers. Three non-synonymous single nucleotide polymorphisms (SNPs) were selected in human *VGF* gene. Transfection of N2a cells with plasmids containing these SNPs revealed no relative change in protein expression levels and normal protein size, except for a truncated protein from the premature stop codon, E525X. All three SNPs resulted in a lower proportion of N2a cells bearing neurites relative to wild-type *VGF*. Furthermore, all three mutations reduced the total length of dendrites in developing hippocampal neurons. Taken together, our results suggest VGF enhances dendritic maturation and that these effects can be altered by common mutations in the *VGF* gene. The findings may have implications for people suffering from psychiatric disease or other conditions who may have altered VGF levels.

## 1. Introduction

The dysregulation of neurotrophins is thought to contribute towards the underlying pathophysiological mechanisms of neurological and neuropsychiatric diseases. VGF (non-acronymic) is a neuropeptide whose expression is upregulated by neurotrophins including brain-derived neurotrophic factor (BDNF) in the hippocampus [1]. VGF and its peptide products [2] have largely been studied for their role in metabolic homeostasis [3,4,5,6,7], pain [8,9,10], neurodegeneration [11,12,13,14] and, more recently, neuropsychiatric disorders [15,16,17]. Several of these diseases are associated with changes in the hippocampus and despite high expression of VGF in this brain region [18,19], the role of this neuropeptide in the hippocampus is not fully elucidated. Major depressive disorder and chronic stress are characterized by downregulation of neurotrophins, such as *BDNF* in the hippocampus [20,21,22]. Similarly, *VGF* was found to be downregulated in the hippocampus in animal models of depression and human bipolar disorder, and previous studies have demonstrated that increasing levels of the VGF-derived C-terminal peptide TLQP-62 results in antidepressant-like behavioral effects in mice [23,24] in a manner that is dependent on BDNF/TrkB/CREB signaling [25]. Parallel to BDNF, TLQP-62’s behavioral actions are also mediated by the PI3K/Akt/mTOR pathway, supporting the notion that VGF is upstream of BDNF signaling [1,26]. Moreover, *VGF* has been shown to be required for the biochemical and behavioral effects of antidepressants [27].

Depression and stress induce remodeling of hippocampal neuronal morphology, including atrophy of neuronal branches, reduction in spines and eventually cell death resulting in a smaller hippocampus [28,29,30]. Studies in animal models have shown that antidepressants, as well as BDNF reverse some of the structural changes to hippocampal neurons induced by chronic stress [31,32,33,34,35,36]. Despite the fact that TLQP-62 acts in concert with both antidepressants and BDNF to affect behavior, the cellular mechanisms within the hippocampus that may underlie TLQP-62’s antidepressant-like effects are only partially understood.

The hippocampus is a critical region in the adult brain involved in the synthesis, maturation, and integration of new neurons and TLQP-62 has been shown to play role in adult neurogenesis, survival, and synaptic plasticity. VGF-derived peptides increase adult neurogenesis in the dentate gyrus [23,27,37,38] and has neuroprotective effects in injured neurons to promote cell survival [39,40]. Studies have shown that neuropeptides are among the many endogenous factors that play a role in reorganizing neuronal networks by modulating neuronal activity [41]. Indeed, *VGF* gene expression is regulated by synaptic activity [19,42,43,44]. Furthermore, similar to BDNF, VGF-derived peptides (TLQP-62) enhance hippocampal synaptic plasticity [1,45]. TLQP-62 peptide is endogenously expressed in the hippocampus and antibodies to TLQP-62 block fear memory training in mice, demonstrating that this C-terminal peptide is critical for learning and memory [46]. In addition to the hippocampus, TLQP-62 peptide has been detected in the hypothalamus and cortex of hamsters using HPLC and ELISA and correlates with the photoperiod [47] whereas, in humans, TLQP-62 is detected in blood and is regulated by glucose metabolism [48]. In behavioral paradigms, *VGF* mutant mice show altered hippocampal-dependent spatial memory formation and reducing VGF levels results in impaired hippocampal-mediated fear conditioning, processes that are dependent on BDNF-TrkB signaling [45,46]. Thus, the role of TLQP-62 in neurogenesis, neuroprotection, and synaptic plasticity are well established.

Synaptic plasticity is influenced by dendrite morphology and synapse formation. Dendrites are sites at which neurons receive information from multiple pre-synaptic partners. Their function is dependent on dendritic branches and small projections called spines which are the postsynaptic site of synapses. The role of VGF in neuronal morphology and dendritic outgrowth has only begun to be explored. VGF has been shown to enhance dendritic growth of multipolar stellate neurons, but not cortical pyramidal neurons [49] and to promote neurite outgrowth of PC12 cells [50]. Although the receptor for the TLQP-62 peptide derived from VGF has not been confirmed, two putative receptors for TLQP-21 have been reported [51,52] and one of them, Complement Protein C1q, has been shown to modulate neurite outgrowth in spinal cord neurons [53]. However, the effects of TLQP-62 on the morphology and maturation of primary hippocampal neurons has not been examined. Moreover, the effect of common polymorphisms in the *VGF* gene [54] on neuronal function have not been explored.

This study explores the neurotrophic effects of VGF, focusing specifically on its actions at the morphological level. We examine the effect of VGF on dendritic and spine morphology in developing primary hippocampal cultures and correlate that to changes in synaptic protein levels and co-localization. We further study VGF’s function by identifying three existing single nucleotide polymorphisms (SNPs) in the human *VGF* gene that are predicted as deleterious and the effect of the SNPs on VGF-induced neurite outgrowth. These studies demonstrate a potential cellular mechanism that VGF may employ to exert its behavioral outcomes.

## 2. Results

### 2.1. TLQP-62 Increases Dendritic Branching and Length but Not the Number of Primary Dendrites

**Figure 1:**
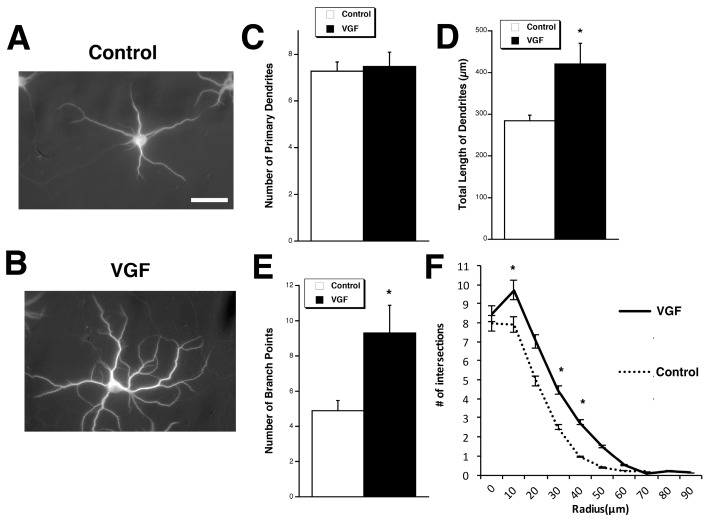
TLQP-62 peptide promotes dendritic branching and length but not number of primary dendrites. (**A,B**) Representative images of hippocampal neurons treated with 3 µM TLQP-62 (VGF) (**A**) or vehicle (control) (**B**) from 4–7 days in vitro (DIV). Scale bar = 60 µm; (**C**–**F**) Quantitation of morphological parameters shows that TLQP-62 has no effect on the number of primary dendrites (**C**) but increases the total length of dendrites (**D**) and the number of branch points as shown by total number of branch points (**E**), as well as Sholl analysis (**F**) (*n* = 35). Bars represent average ± SEM. Two-sample *t* test of control vs. VGF: (**C**) Number of primary dendrites t(6) = 0.17, *p* = 0.787; (**D**) total length of dendrites t(6) = 1.38, *p* = 0.037; (**E**) number of branch points t(6) = 1.40, *p* = 0.039; (**F**) 10 µm t(6) = 0.32, *p* = 0.60; 20 µm t(6) = 1.35, *p* = 0.042; 30 µm t(6) = 0.82, *p* = 0.182; 40 µm t(6) = 1.40, *p* = 0.050; 50 µm t(6) = 1.33, *p* = 0.043; 60 µm t(6) = 1.22, *p* = 0.056; 70 µm t(6) = 1.01, *p* = 0.107. * *p* < 0.05, unpaired *t* test.

To explore the potential effects of VGF on dendritic outgrowth, developing primary hippocampal cultures at four days in vitro (DIV) were exposed to VGF peptide, TLQP-62 (3 µM), for three days, after which morphological parameters were measured in pyramidal cells. Compared to control, exogenous TLQP-62 significantly increased the total primary dendritic length and number of branch points while preserving the overall number of primary dendrite (Figure 1A–E). Sholl analysis revealed an increase in dendritic branching in these developing neurons (Figure 1F).

### 2.2. AAV VGF Increases the Number of Immature Spines in Primary Hippocampal Cultures

**Figure 2:**
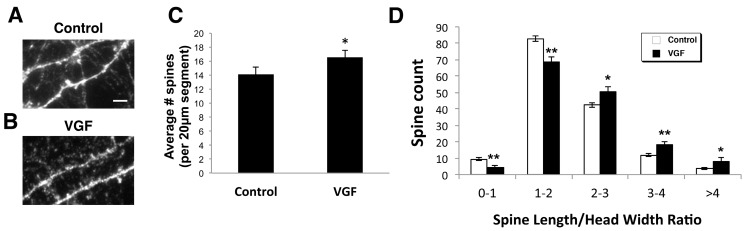
VGF overexpression increases immature spine formation. (**A**,**B**) Representative images of AAV infected hippocampal cultures (14–20 DIV) treated with AAV-GFP (control) (**A**) or AAV-VGF-GFP (VGF) (**B**); The signal was enhanced with GFP immunohistochemistry. Scale bar = 5 µm; (**C**) Quantification of spine density. Bars represent average number of spines per 20 µm dendritic segment ± SEM (*n* = 6 dishes, 15 segments/dish); (**D**) quantitation of spines in Green Fluorescent Protein (GFP) or VGF (non-acronymic) treatment groups based on ratio of spine length/head width ± SEM (*n* = 4 dishes, 225 spines/dish). Two-sample *t*-test of control vs. VGF: (**C**) average number of spines t(22) = 4.66, *p* = 1.72 × 10^−7^; (**D**) spine length/head width ratio 0–1 t(14) = −3.04, *p* = 0.003; 1–2 t(14) = 2.25, *p* = 0.002; 2–3 t(4) = 2.45, *p* = 0.032; 3–4 t(14) = 2.25, *p* = 0.008; >4 t(14) = 2.25, *p* = 0.038. * *p* < 0.05, ** *p* < 0.01 relative to control (unpaired *t*-test).

In addition to examining VGF’s large-scale effects on dendritic branching and length, we also assessed potential changes in dendritic spines, the site of fine-scale synaptic tuning. Primary hippocampal cultures (14 DIV) were infected with a *VGF*-encoded Adeno Associated Virus (AAV-vector) along with green fluorescent protein (GFP), to overexpress VGF. This delivery system provides the opportunity for a more physiological response to VGF than adding VGF exogenously because it allows cells to synthesize the protein and for endogenous proteases to generate the relevant mature peptide(s). AAV-VGF-GFP cultures showed a significant increase in dendritic spine density compared to control-treated AAV-GFP cultures (Figure 2A–C).

To further characterize the maturity of the spines, we also measured spine length and head width and binned the spine length/head width ratio into different groupings. The spine types that VGF significantly increased were spines with larger spine length/head width ratios (Figure 2D), which signifies that VGF increased the number of longer, thinner spines, a phenotype consistent with immature spines.

### 2.3. TLQP-62 Peptide Increases Levels of Synaptic Proteins and Number of Functional Synapses In Vitro

**Figure 3:**
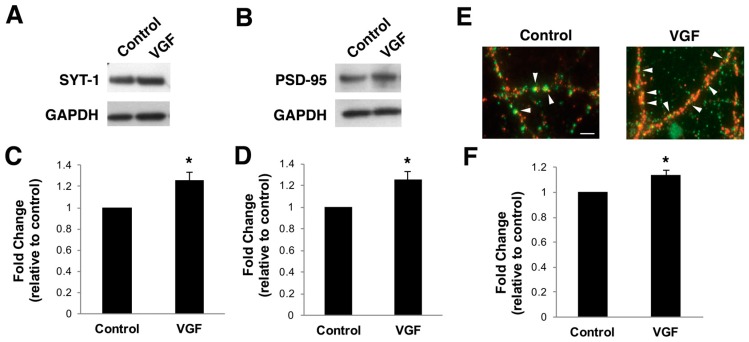
TLQP-62 peptide enhances the levels of synaptic proteins and synaptic contacts. (**A**,**B**) Representative Western blot of hippocampal cultures (14 DIV) that had been pretreated with vehicle or TLQP-62 (3 µM) for three days. Protein lysates were run on a 4%–12% NuPAGE Tris gel and the membrane probed for either synaptotagmin (SYT-1) (**A**) or PSD-95 (**B**) followed by GAPDH for loading control; (**C**) quantitation of SYT-1 protein expression. Bars represent average protein expression compared to control ± SEM (*n* = 7); (**D**) Quantitation of PSD-95 protein expression. Bars represent average protein expression compared to control ± SEM (*n* = 6) * *p* < 0.05 relative to control (unpaired *t*-test); (**E**) representative images of immunostained hippocampal cultures (17 DIV) treated with vehicle or TLQP-62 (3 µM) for three days. The cultures were probed for synaptotagmin (SYT-1) (**red**) and post-synaptic protein PSD-95 (**green**). Points of co-localization are indicated by white arrowheads. Scale bar = 10 µm; (**F**) quantitation of co-localization are shown in bar graph. Synapses were counted on three 50 µm secondary dendrite segments per neuron (*n* = 60). Bars represent average synapses per dendrite relative to the control of each set ± SEM (*n* = 4). Two-sample *t*-test of control vs. VGF: (**C**) SYT-1 t(18) = 1.37 *p* = 0.014, (**D**) PSD-95 t(10) = 1.57 *p* = 0.011, and (**E**) synapses t(148) = 1.30 *p* = 0.010. * *p* < 0.05 relative to control (unpaired *t*-test).

We next examined if the newly-formed spines are forming synapses. To assess VGF’s effects on synaptic protein expression, lysates from primary hippocampal cultures (17 DIV) treated with exogenous TLQP-62 peptide (3 µM) for three days were probed for pre-and post-synaptic proteins, synaptotagmin-1 (SYT-1) and postsynaptic density-95 (PSD-95), respectively. Both PSD-95 and SYT-1 were significantly upregulated in TLQP-62-treated cultures compared to the control (Figure 3A–D).

Immunohistochemistry was performed on similarly treated cultures to visualize the localization of increased synaptic protein expression in VGF-treated cultures. TLQP-62 significantly increased the amount of co-localization of SYT-1 and PSD-95 compared to control conditions (Figure 3E,F), indicating an increase in the number of synaptic sites of contact and suggesting an upregulation of functional synapses.

### 2.4. Identification of Potential Deleterious Non-Synonymous Missense and Nonsense VGF Single Nucleotide Polymorphisms and Their Effects on Protein Expression

**Figure 4:**
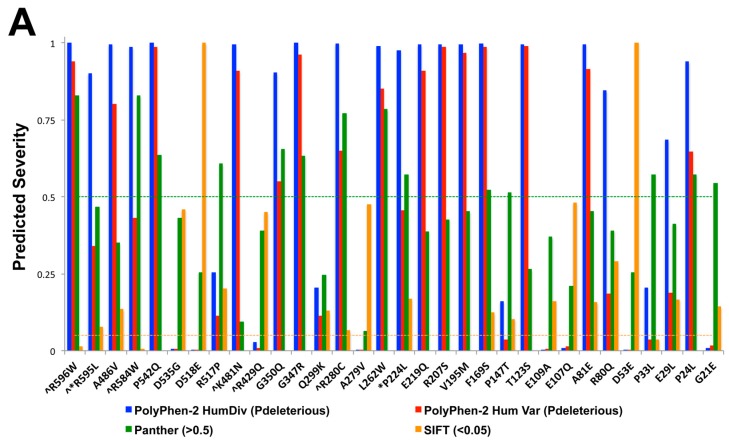

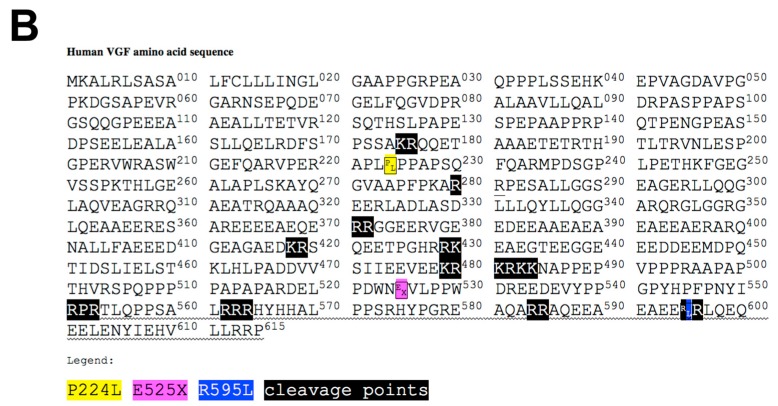
Predicted severity and location of nonsynonymous missense single nucleotide polymorphisms in *VGF*. (**A**) Three programs were used to identify possible deleterious mutations including PolyPhen-2 Hum Div (**blue**) PolyPhen-2 Hum Var (**red**), Panther (**green**), and SIFT (**gold**). Criteria were a P deleterious for Polyphen-2, Panther value > 0.5, and SIFT value < 0.05. < indicates peptide cleavage site. * indicates SNP pursued in this study. (**B**) Human VGF amino acid sequence. The location of SNPs are indicated by color: yellow = P224L, purple = E525X, blue = R595L, black = cleavage points.

Since VGF precursor is proteolytically cleaved into several smaller neuropeptide fragments with various biological functions, it is important to characterize the effects of single base pair variations that may affect protein integrity and function. To identify suitable SNPs of interest, results from an NCBI dbSNP query search for known existing SNPs found within the *VGF* reading frame were compiled, and their predictive effects analyzed using three different theoretical modeling softwares [Sorting Intolerant from Tolerant (SIFT), Panther, Polyphen] (Figure 4A). Plasmids containing clones from *VGF* SNPs with a predictive deleterious base-pair variation in each of the three programs were created, including proline to leucine at amino acid 224 (P224L), found in the middle region of the protein, glutamic acid to a stop codon at amino acid 525 (E525X), a premature stop codon, and arginine to leucine at amino acid 595 (R595L), found in the C-terminal region, which is thought to be the active moiety (Figure 4B).

**Figure 5:**
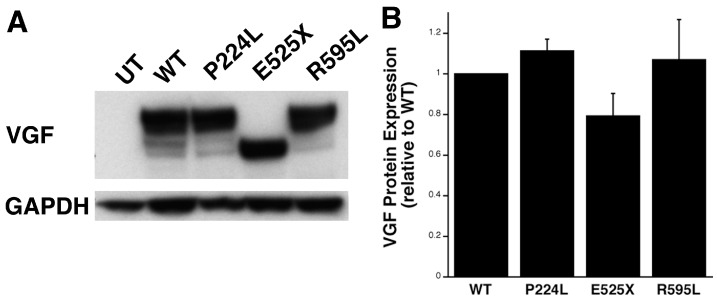
*VGF* Single Nucleotide Polymorphism (SNP) E525X results in truncated protein. (**A**) Representative Western blot of protein lysates from Human Endothelial Kidney (HEK) cells transfected with *VGF* plasmid containing either a mutation representing the P224L, E525X or R595L SNPs or Wild Type (WT) *VGF*, or Untransfected (UT). The blot was probed with VGF antibody and GAPDH was used as a loading control; and (**B**) quantification of protein expression for VGF plasmids in HEK cells. Bars represent average VGF protein levels normalized to GAPDH and expressed as a fold change relative to WT ± SEM. (*n* = 3). Two-sample *t* test: WT vs. P224L t(4) = 2.04, *p* = 0.125. WT vs. E525X t(4) = −1.80, *p* = 0.137. WT vs. R595L t(4) = 0.386, *p* = 0.740.

The effect of the SNPs on VGF protein expression was determined by transfecting HEK-293 cells with plasmids generated by site-directed mutagenesis to modify the *VGF* gene to contain each one of the SNPs identified above. Lysates from a transfected HEK-293 cell line overexpressing plasmids containing *VGF* SNPs were collected and probed for VGF expression. No differences in overall VGF protein expression between wild-type and each of the three SNPs were detected. As predicted, E525X, which corresponds to a premature stop codon, exhibited a truncated protein product, while the other two SNPs were no different in size compared to the WT *VGF* plasmid (Figure 5).

### 2.5. VGF SNPs Reduce Process Outgrowth in N2a and Primary Hippocampal Cultures

**Figure 6:**
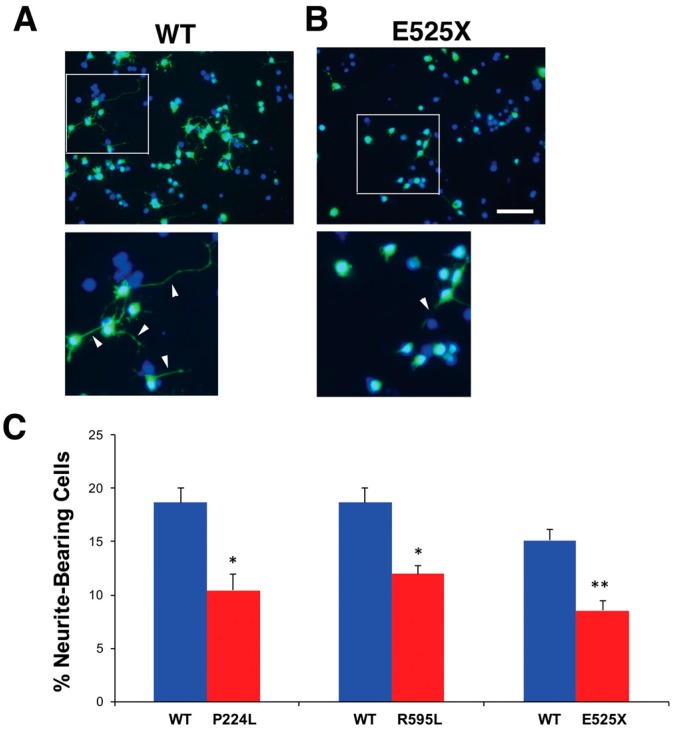
N2A cells transfected with *VGF* SNP plasmids exhibit fewer neurites. (**A**,**B**) Representative images of N2A cells expressing plasmid containing either WT *VGF* (**A**) or E525X mutation in VGF (**B**) and stained for GFP (**green**) and DAPI (**blue**). Higher magnification view of boxed area is shown below. Neurites are visible extensions from the cell body as indicated by white arrowheads. Scale bar = 100 µm in top images and 60 µm in bottom; and (**C**) quantification of neurite extension from N2A cells. Bars represent average percent neurite-bearing cells ± SEM. 150 cells/dish were counted (*n* = 3), Two-sample *t* test: WT vs. P224L t(4) = −2.24, *p* = 0.015. WT vs. R595L t(4) = −2.21, *p* = 0.014. WT vs. E525X t(8) = −3.40, *p* = 0.002. * *p* < 0.05, ** *p* < 0.01, unpaired *t*-test.

To begin to address the potential functional significance of *VGF* SNPs, neuroblastoma N2a cells were transfected with plasmids containing the SNPs, cultured for 72 h, fixed and then their process outgrowth analyzed. All three *VGF* SNP-transfected cultures featured a significant decrease in the percent of cells bearing processes for each of the SNP plasmids relative to WT *VGF* plasmids (Figure 6).

**Figure 7:**
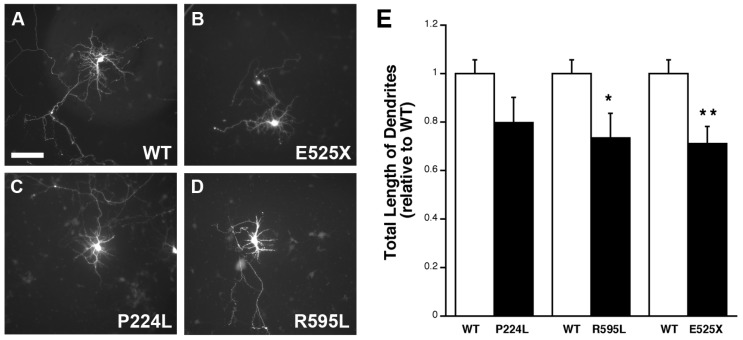
Hippocampal cells transfected with *VGF* SNP plasmid exhibit shorter neurites. (**A**–**D**) Representative images of hippocampal cells transfected with plasmids containing either WT VGF (**A**) or *VGF* SNPs E525X (**B**), P224L (**C**), R595L (**D**) from 4–7 DIV, Scale bar = 80 µm; and (**E**) bar graphs represent average total length of dendrites normalized to WT neurons ± SEM as quantified using NeuronJ. (*n* = 7, 8), Two-sample *t* test: WT vs. P224L t(17) = −0.166, *p* = 0.114. WT vs. R595L t(16) = −0.789, *p* = 0.038. WT vs. E525X t(12) = −1.36, *p* = 0.006. * *p* < 0.05, unpaired *t*-test, ** *p* < 0.01, unpaired *t*-test.

The effect of the SNPs on dendritic outgrowth and morphology was also assessed in developing primary hippocampal neurons. Cultures (4 DIV) were similarly transfected with WT or mutant plasmids and analyzed for differences in the number of primary dendrites, number of branch points and average total length of dendrites. While the first two parameters remained no different between groups (data not shown), *VGF* SNP-transfected cells exhibited a significantly shorter average total dendrite length compared to wild-type for E525X and R595L and a trend to lower dendritic length for P224L (Figure 7). Together, this data demonstrates that these particular *VGF* SNPs mitigate VGF’s effects on potentiating neurite outgrowth, and may suggest underlying circuitry impairments within individuals who possess such SNPs.

## 3. Discussion

BDNF and NGF are well-known neurotrophins involved in neuronal development and synaptic remodeling, yet their mechanisms of action and downstream mediators continue to be further characterized. The present study examined the neurotrophin-inducible neuropeptide VGF, which has been identified as an antidepressant-like agent [23,24], as well as a key player in learning and memory [1,45,46]. We observed that VGF-derived peptide TLQP-62 exhibited morphologic changes, such as promoting dendritic outgrowth, immature spine formation, and synaptogenesis, in primary hippocampal cultures. Although many of the effects we observed for TLQP-62 on hippocampal neuronal morphology and maturation are similar to what has been reported for BDNF, there are a few noticeable differences. For example, BDNF has been shown to enhance primary neurite numbers, as well as dendritic outgrowth and branching [34,55], whereas we demonstrate that TLQP-62 does not affect the number of primary dendrites and only promotes dendritic length and secondary branching. Our data, thus, suggest that TLQP-62 may have more of a role during the later stages of dendritogenesis rather than the initial formation of dendritic outgrowths. Contrary to its effects in hippocampal cultures, the overexpression of VGF has been shown to increase primary neurites in PC12 cells [50]. Our previous studies have demonstrated that TLQP-62 induces the ERK, Akt and GSK3β signaling pathways in hippocampal cells [27,56] which have been shown to be involved in neurite outgrowth [57], however the signaling pathways activated by TLQP-62 in PC12 cells have not been studied. Furthermore, since VGF is processed into several bioactive peptides [2], it is possible that alternative peptides are generated in different cell types and exert varying effects on neuronal morphology. For example, it would be of interest to examine if TLQP-21 which is a shorter peptide consisting of a subset of TLQP-62 also has the same effects on morphology. Moreover, future experiments examining the effects of the different VGF peptides on cytoskeletal components may further reveal the mechanism by which VGF promotes dendritic branching.

In addition to showing that TLQP-62 has gross morphological effects on dendritic arborization, we also demonstrated that VGF overexpression increases spine density and induces the generation of immature spines in primary hippocampal cultures. These findings are also in contrast to BDNF, which has been primarily shown to promote the formation of mature, stubby spines [34,58], although one study demonstrates that a TrkB agonist increases thin spine density in a mouse model of Alzheimer’s Disease [59]. Spines are the site of fine-tuning synaptic plasticity, whose induction is much faster than that of large-scale dendritic outgrowth. Activity-regulated cytoskeletal protein (Arc) is a BDNF-regulated immediate early gene has been shown to be instrumental in spine dynamics critical for LTP and learning [60]. We have previously shown that Arc is co-regulated with VGF in hippocampal neurons and that TLQP-62 promotes synaptic plasticity in an acute fashion [1]. Arc potentiates the formation of thin spines, in an AMPA-endocytosed, mediated manner [61]. The thinner dendritic spines seen in response to VGF treatment may therefore correspond to a more plastic state. An additional point to address is whether upon stimulation, these highly plastic spines possess a greater ability to develop into more mature, mushroom-shaped spines [34]. Cultures exposed to TLQP-62 exhibited a greater expression and co-localization of SYT-1 and PSD-95, pre- and post-synaptic markers of functional synapses which is consistent with studies correlating VGF expression with the timing of synaptogenesis in developing neurons [62,63]. Taken together, our data support the idea that VGF promotes the formation of immature spines which are primed form a synapse with a pre-synaptic bouton.

An additional point the present study sought to understand is the consequence of *VGF* single nucleotide polymorphisms on protein integrity and function. To date it is not known if *VGF* polymorphisms contribute towards pathogenesis or treatments of psychiatric disorders or neurodegenerative disease. Our investigation revealed impaired dendritic outgrowth in cultures treated with mutant VGF protein corresponding to known existing SNPs. Although the data presented in this study are all in vitro, as a potential targeted common pathway for antidepressant therapies and in neurodegenerative disorders, it will be critical in future studies to understand the effect of single nucleotide polymorphisms on therapy response. This is especially important given that 30% of patients currently treated with selective serotonin re-uptake inhibitors (SSRIs) are classified as non-responders, which may correspond to impaired downstream mechanisms otherwise targeted in responders. Our assessment of several known *VGF* SNPs demonstrated impaired dendritic outgrowth in N2a and primary hippocampal cultures. This maladaptive response may cause deficits in neural circuitry formation, neurogenesis, and impaired synaptic plasticity given the important role that TLQP-62 has been shown to play in neurogenesis [23,27,37], synaptic plasticity [1], and LTP [45]. Furthermore, dysfunctional VGF proteins may cause a blunted response to antidepressants and additional therapies. Given the small prevalence of individuals with these *VGF* SNPs, it remains unknown whether these individuals are at an increased risk for neuropsychiatric disorders. Current evidence suggests that individuals with the BDNF Val66Met polymorphism (Rs6265), which includes 25% of the population, are at an increased risk of developing either an anxiety or depressive-related disorder. Furthermore, depressed patients with this SNP show a preferential response to tricyclic antidepressants (TCA) and serotonin and norepinephrine reuptake inhibitors (SNRIs) over SSRIs [64,65]. Determining the mechanisms that account for preferential differences in treatment response will enable future treatment strategies to exploit alternative pathways and molecular players in subsets of patients who do not respond to conventional medications. Moreover, since VGF has diverse roles in the hippocampus, including learning and memory [1,45,46], and is associated with impaired memory and Alzheimer’s Disease [11,12,66,67], it will also be important in future studies to explore the role of the *VGF* SNPs in neurodegenerative diseases as has been done for Val66Met *BDNF* polymorphism [68,69].

While there is no definitive consensus by which antidepressants work, prevailing theories suggest that therapies target maladaptive synaptic circuitry [70]. Regions implicated in neuropsychiatric disease that are of particular importance include the hippocampus, a limbic structure involved in learning, memory, and stress response, and the prefrontal cortex, which has vast connections with monoaminergic- and mood-regulating areas. A plethora of evidence shows gross perturbations in postmortem depressed patients, specifically within the stress-sensitive hippocampus where reports document atrophy, which may result from thinning of spines, reduced dendritic arborization, and cell death [71,72,73]. Moreover, depressed and bipolar patients exhibit downregulated neural plasticity-related and neuroprotective proteins, including BDNF, within these key regions [74]. Recent clinical data has shown that effective responders to antidepressants experience an improvement in neural plasticity-related gene expression, including BDNF and VGF, a finding absent in non-responders [17,75].

Although typical antidepressants (TCAs, SSRIs, SNRIs) and atypical therapies (including electroconvulsive shock and ketamine) differ in their onset of therapeutic action, they share similar downstream signaling cascades, indicating conserved therapeutic mechanisms [76,77]. Like BDNF, VGF has been shown to be one such common denominator amongst antidepressants, implicated in the effects of typical, fast-acting atypical, and mood enhancing agents (e.g., lithium and valproic acid) [26,27]. The delayed therapeutic onset commonly associated with classic antidepressants coincides with the upregulation of BDNF, and presumably downstream effectors, such as VGF, which happens much sooner in the case of fast-acting atypical antidepressants, like ketamine [78]. Our most recent findings demonstrate that TLQP-62 potentiates dendritic lengthening and secondary branching while preserving primary branch points, which follows a similar pattern of the aforementioned therapeutic agents. In particular, lithium and valproic acid have been shown to promote lengthening of primary hippocampal neurites [79]. There is evidence in the literature that other neuropeptides can influence neuronal morphology including pituitary adenylate cyclase-activating peptide (PACAP) [22], Neuritin [80], Nociceptin [81], and vasoactive intestinal peptide [82]. Furthermore, several of these neuropeptides have also been shown to have antidepressant-like effects [83,84] similar to TLQP-62. Previous work from our lab revealed that VGF signaling is critical for the molecular and behavioral effects of lithium. In particular, VGF heterozygotes performed worse than their wildtype counterparts in a novelty-induced hypophagia model, which may be explained by impaired lithium-induced ERK/MAPK and Akt-mediated GSK 3β phosphorylation [27]. Together, this suggests that lithium’s effects on dendritic outgrowth are in part mediated via VGF.

Previously published work from our lab revealed the mechanisms involved in TLQP-62 -induced neurogenesis [27,37], another process by which the neuropeptide may mediate its antidepressant-like behavioral effects and we are currently exploring if TLQP-62-induced neurogenesis is also affected by SNPs. We have shown that TLQP-62-induced neurogenesis is dependent on NMDA and metabotropic GluR5 receptors, as well as their downstream mediators, calcium/calmodulin dependent protein kinase II (CaMKII) and protein kinase D (PKD), respectively [37]. Given that these same signaling cascades have been implicated in dendritic outgrowth and synaptic plasticity [85,86], TLQP-62 may also act through these pathways to effect the morphological changes in neurons. By continuing to explore the cellular effects of VGF on hippocampal neurons, the mechanisms underlying the behavioral actions of VGF may be revealed and novel treatment approaches to various diseases in which VGF has been implicated may be developed.

## 4. Materials and Methods

### 4.1. Ethics Statement

The procedures described were conducted in accordance with the National Institutes of Health (NIH) guidelines and were approved by the Institutional Animal Care and Use Committee at Rutgers University. The protocol number I12-006 was approved on 12/19/2016. The Rutgers–Robert Wood Johnson Medical School assurance number with the Office of Laboratory Animal Welfare is A3328-01.

### 4.2. Hippocampal Cultures

E18 (embryonic day 18) hippocampi were obtained from timed-pregnant Sprague Dawley rats (Charles River, Wilmington, MA, USA) killed by CO_2_ asphyxiation. Pooled tissue from each litter was mechanically triturated in Eagle’s Minimum Essential Medium (MEM) with glucose and 7.5% fetal bovine serum and plated on poly-d-lysine-coated petri dishes at 350,000 cells/35 mm dish. Cultures were maintained in serum-free neurobasal medium containing B27 (Invitrogen, Grand Island, NY, USA) and glutamine at 37 °C in a 95% air/5% CO_2_ humidified incubator as previously described [87] and contained virtually pure neurons.

### 4.3. Dendritic Branching

Hippocampal cell cultures at four days in vitro (DIV) were treated with 3 µM TLQP-62 (TLQP-62 C-terminal amidated peptide) (Biopeptide, San Diego, CA, USA) or dH_2_O control from four days in vitro (DIV) until 7 DIV. At 7 DIV the cells were fixed in 4% paraformaldehyde (PFA) for 15 min. For visualization of dendrites, monoclonal anti MAP2ab primary antibody (1:1000, Sigma, St. Louise, MO, USA) was applied overnight at 4 °C. Secondary antibody, AlexaFluor 594 goat anti-mouse (1:1000, Invitrogen) was then added and incubated for 1 h at room temperature. Cells were cover-slipped with Fluoromount-G (Southern Biotech, Birmingham, AL, USA). Neurons were imaged on a Zeiss microscope at 40× and analyzed by Neuro-Lucida software (Version 11.08.2, MBF Bioscience, Willston, VT, USA).

### 4.4. Dendritic Spines

VGF was overexpressed in hippocampal cell cultures using adeno-associated virus (AAV) vectors tagged with GFP (Vector BioLabs, Eagleville, PA, USA). Hippocampal neuronal cultures were treated with VGF-GFP or control GFP virus at 14 DIV. Each culture dish was treated with 20 µL of either virus (VGF-GFP or GFP) for 6 days at final concentrations of 2.45 × 10^9^ GC/mL for the VGF-GFP virus and 9.80 × 10^8^ for the GFP control virus in Neurobasal medium. Dishes were then fixed in 4% PFA at 20 DIV. Immunostaining was performed in order to enhance the GFP fluorescence. Cells were incubated in chicken anti-GFP (1:500, Invitrogen) overnight at 4 °C. Secondary antibody, rabbit anti-chicken FITC (1:500, Invitrogen) was applied for 1 h at room temperature. The dishes were slipped with Fluoromount-G. Spine morphology observation was performed with a confocal microscope with the Fluoview program. Dendrites were imaged with a 60× lens and 2× digital zoom. Spines were measured in ImageJ software for head width and spine length. Head width was measured as the distance from the rightmost side of the largest part of the spine to the leftmost side. Spine length was measured as the distance from the top of the spine to its base. For dendritic spine density, spines were counted per 20 µM dendritic segment in Axiovision software (Version 4.8.2, Zeiss, Jena, Germany).

### 4.5. Synaptic Protein Immunostaining

Hippocampal cell cultures were treated at 17 DIV with 3.0 µM TLQP-62 or dH_2_O control until 20 DIV. Following fixation with cold methanol, primary antibodies for presynaptic marker synaptotagmin (1:200, Cell Signaling 3347, Danvers, MA, USA) and postsynaptic marker PSD-95 (1:200, Affinity Bioreagents MA1-045, Golden, CO, USA) were added simultaneously and incubated overnight at 4 °C. Secondary antibodies were goat anti-rabbit AlexaFluor594 (1:1000, Invitrogen) for synaptotagmin and goat anti-mouse AlexaFluor488 (1:200, Invitrogen) for PSD-95 which were applied for 1 h at room temperature. Cells were cover-slipped in Fluoromount-G. Synapses were quantitated at 63× using a Zeiss microscope by measuring the number of co-localized green and red puncta. Three secondary dendrites were analyzed per neuron with 15 neurons per treatment group. Each dendrite segment was approximately 50 µM in length.

### 4.6. Synaptic Protein Western Blots

Hippocampal cell cultures were treated at 17 DIV with 3.0 µM TLQP-62 or dH_2_O control until 20 DIV. Cells were washed twice with cold phosphate-buffered solution (PBS) and then treated with 100 µL lysis buffer made from mammalian protein extraction reagent (M-PER), ethylenediaminetetraacetic acid (EDTA) (10 µL/mL) and inhibitor cocktail (10 µL/mL) (Thermo Scientific, Rockford, IL, USA). Cells lysates were collected and centrifuged at 4 °C at 10,000 rpm for 5 min. The supernatant was transferred to Amicon Ultra (3000 MW) tubes and centrifuged for 10 min at 10,000 rpm. The total amount of protein in the concentrate was quantified using Pierce BCA reagent (Thermo Fisher Scientific, Waltham, MA, USA). 30 µg of the protein lysate was then loaded onto a 4%–12% Nu-polyacrylamide gel electrophoresis (NuPAGE)-Tris gel and transferred onto a polyvinylidene difluoride membrane. Primary antibody, PSD-95 (1:2000, Affinity Bioreagents MA1-045) or synaptotagmin (1:2000, Cell Signaling) was then added and incubated overnight at 4 °C. The membranes were then treated with donkey anti-rabbit or anti-mouse horseradish peroxidase-conjugated IgG (1:5000, GE Healthcare, Pittsburgh, PA, USA) for 1 h at room temperature. Proteins were detected using enhanced chemiluminescence (PerkinElmer, Waltham, MA, USA). Levels of the immunopositive bands were quantified densitometrically using Quantity One V 4.2.1 software on a GelDoc 2000 (BioRad, Hercules, CA, USA) [87]. Levels of protein expression were quantitated and normalized to glyceraldehyde-3-phosphate dehydrogenase (GAPDH) levels (1:1000, Meridian Life Science Inc., Cincinnati, OH, USA).

### 4.7. SNP Structure and Function Prediction 

Three separate programs [Sorting Intolerant from Tolerant (SIFT), Panther and Polyphen-2] were used to predict the significance of several known existing VGF SNPs from the NCBI Database on protein structure and function. Polyphen-2 was used to search for the query protein in the UniprotKB/Swiss-Prot database; this determined whether the amino acid change occurs within an important region as determined by evolutionary conservation region that is important (i.e., disulfide bridge, ligand binding site). It then maps the change to a known 3-D structure to determine its effect [88]. Panther also predicts the effect of non-synonymous SNPs on protein function via the calculation of a substitution position-specific evolutionary conservation (subPSEC) score based on the alignment of the sequence in question with a library of evolutionarily related proteins [89]. Panther utilizes what it describes as a “PANTHER Library” and a “PANTHER Index”. The library consists of collections of protein families, organized as multiple sequence alignments, hidden Markov models, and family trees. The index summarizes the various functions and processes associated with the individual protein families and subfamilies. By utilizing the Hidden Markov Models, Panther is able to rank SNPs in terms of their potential effect on protein function. The SIFT algorithm is based on evolutionary conserved sites within protein and uses PSI-BLAST to predict the effect of amino acid substitutions on protein function by comparing the query sequence with other closely related sequences [90].

### 4.8. VGF SNP Plasmid Site-Directed Mutagenesis

Primers specific to respective SNPs of interests [P224L (rs151121493), E525X (rs35400704), R595L (rs201441915)] were used with the Quick Lightning Site-Direct Mutagenesis Kit (Agilent, La Jolla, CA, USA) to induce single-base pair point mutations in the VGF Human cDNA ORF Clone (rc209477, OriGene, Rockville, MD, USA). Betaine (1 M) and DMSO (5%) were used as PCR additives to minimize potential secondary structure in CG-rich areas. Template was then digested with DpnI to eliminate parental methylated and hemimethylated DNA. All SNP-containing plasmids were transfected into competent *Escherichia coli* cells. Sequencing was performed to confirm creation of SNPs (Genewiz, South Plainfield, NJ, USA).

### 4.9. Analysis of SNPs in HEK cells

HEK 293 cells (ATCC, Manassas, VA, USA) were cultured in DMEM culture media containing 10% FBS and 1% Pennicillin Streptomycin (Pen Strep). At 70% confluency, cells were transfected with Lipofectamine 2000 Reagent (Invitrogen) and 15 µg of plasmid containing one of the VGF SNPs. Transfection efficiency per culture plate was determined using co-transfection with green fluorescent protein (GFP) plasmids and was very similar between dishes. Two days following transfection, HEK-293 cells were solubilized in radioimmunoprecipitation assay buffer (RIPA) buffer and protein was determined by bicinochoninic acid (BCA) assay (ThermoFisher Scientific). Forty micrograms of protein was run on a 10% Bis-Tris gel and probed with anti-VGF (R-15, sc-365397) (1:500, Santa Cruz Biotechnology, Santa Cruz, CA, USA) and anti-GAPDH (1:1000, Meridian Life Science Inc.). Secondary antibodies were anti-mouse HRP (1:5000, GE Healthcare Life Sciences) followed by enhanced chemiluminescent (ECL) detection (Perkin Elmer). Detection and quantitation was performed on a FluorChem HD2 chemiluminescence detection machine (Protein Simple, San Jose, CA, USA).

### 4.10. SNP Effect on N2A Cell Process Outgrowth

Mouse N2a cells (ATCC) were cultured in T-75 flasks with DMEM (high glucose, with l-glutamine) containing 10% FBS, 1% Pen Strep incubated at 37 °C in a 95% air/5% CO_2_ incubator and split at 70%–90% confluency. Prior to transfections, cells were seeded at 2.25 × 10^5^ cells/35 mm Nunclon plates under serum and antibiotic free conditions. Cultures were then transfected the same day using Lipofectamine 2000 Reagent (Invitrogen) and 1 µg plasmid containing one of the VGF SNPs and 1 µg GFP plasmid (Vector BioLabs). Transfection efficiency per culture plate was determined using GFP expression and was very similar between dishes. Medium was replaced with Neurobasal containing 1% B27 and 1% Pen Strep. Three days following the transfection, cells were fixed in cold 4% PFA, incubated with 4’,6’-Diamidino-2-Phenylindole (DAPI) (1:1000) for 5 min and cover-slipped. Cells bearing processes with a length >2 cell body diameters were deemed neurite-bearing.

### 4.11. SNP Effect on Hippocampal Neurite Outgrowth 

Hippocampal culture neurons at 4 DIV were transfected with wildtype VGF or VGF SNP plasmids using Lipofectamine 2000 (ThermoFisher Scientific). Cells were first washed with Neurobasal and glutamine media and left in Neurobasal, glutamine, and B27 media. One microgram per milliliter of plasmid and GFP, and 1 µL/mL of the Lipofectamine reagent were incubated in Neurobasal and glutamine media for 5 min. Lipofectamine reagent (2.5 µL/mL) was then added to the plasmid complex and incubated at room temperature for 30 min. The whole plasmid complex was added to the cells and incubated for 5 h. Following the transfection, the cells were left in media containing Neurobasal, glutamine, B27, and Pen Strep. Transfection efficiency per culture plate was determined using GFP expression and was very similar between dishes. Three days post transfection, the cells were fixed with 4% PFA. To further enhance GFP signal, immunostaining was performed as described for spines above. Images of neurons were captured at 20× using a Leica microscope (Model DMIRB, Leica Microsystems, Buffalo Grove, IL, USA) and analyzed with ImageJ (Version 1.48, National Institutes of Health, Bethesda, MD, USA).

### 4.12. Data Analysis

Statview software (Version 5.0.1, SAS, Cary, NC, PA, USA) or Excel StatPlus (Version 6.1.1.0, Analyist Soft, Walnut, CA, USA) was used for analysis of all data. Data were analyzed using unpaired, two-tailed Student’s *t*-test. *p* < 0.05 is considered significant.
